# Diagnostics and monitoring tools for noncommunicable diseases: a missing component in the global response

**DOI:** 10.1186/s12992-021-00676-6

**Published:** 2021-03-09

**Authors:** Antonio Bernabé-Ortiz, Jessica H. Zafra-Tanaka, Miguel Moscoso-Porras, Rangarajan Sampath, Beatrice Vetter, J. Jaime Miranda, David Beran

**Affiliations:** 1 CRONICAS Centre of Excellence in Chronic Diseases, Universidad Peruana Cayetano Heredia, Lima, Peru; 2 School of Medicine “Alberto Hurtado”, Universidad Peruana Cayetano Heredia, Lima, Peru; 3 Foundation for Innovative and New Diagnostics, Geneva, Switzerland; 4 The George Institute for Global Health, UNSW, Sydney, Australia; 5 Division of Tropical and Humanitarian Medicine, University of Geneva and Geneva University Hospitals, Geneva, Switzerland

**Keywords:** Noncommunicable diseases, Primary Health Care, Diagnostics

## Abstract

A key component of any health system is the capacity to accurately diagnose individuals. One of the six building blocks of a health system as defined by the World Health Organization (WHO) includes diagnostic tools. The WHO’s Noncommunicable Disease Global Action Plan includes addressing the lack of diagnostics for noncommunicable diseases, through multi-stakeholder collaborations to develop new technologies that are affordable, safe, effective and quality controlled, and improving laboratory and diagnostic capacity and human resources. Many challenges exist beyond price and availability for the current tools included in the Package of Essential Noncommunicable Disease Interventions (PEN) for cardiovascular disease, diabetes and chronic respiratory diseases. These include temperature stability, adaptability to various settings (e.g. at high altitude), need for training in order to perform and interpret the test, the need for maintenance and calibration, and for Blood Glucose Meters non-compatible meters and test strips. To date the issues surrounding access to diagnostic and monitoring tools for noncommunicable diseases have not been addressed in much detail. The aim of this Commentary is to present the current landscape and challenges with regards to guidance from the WHO on diagnostic tools using the WHO REASSURED criteria, which define a set of key characteristics for diagnostic tests and tools. These criteria have been used for communicable diseases, but so far have not been used for noncommunicable diseases. Diagnostic tools have played an important role in addressing many communicable diseases, such as HIV, TB and neglected tropical diseases. Clearly more attention with regards to diagnostics for noncommunicable diseases as a key component of the health system is needed.

## Introduction

A key component of any health system is the capacity to diagnose individuals with a given condition. One of the six building blocks of a health system as defined by the World Health Organization (WHO) includes diagnostic tools. Specifically, for noncommunicable diseases (NCD), the WHO’s Noncommunicable Disease Global Action Plan (NCD GAP) includes addressing the lack of diagnostics through multi-stakeholder collaborations to develop new technologies that are affordable, safe, effective and quality controlled, and improving laboratory and diagnostic capacity and human resources [1]. Specifically, the NCD GAP includes a target of: “an 80 % availability of the affordable basic technologies and essential medicines, including generics, required to treat major noncommunicable diseases in both public and private facilities” [1], which should be ensured.

In 2018, the WHO published the first edition of the Model Essential Diagnostics List (EDL) which was updated in 2019 [2]. The EDL, similar to the WHO Model List of Essential Medicines (EML), was aimed at ensuring essential diagnostics are available for the achievement of Universal Health Coverage (UHC). As with the EML, the EDL aims to serve as a guiding document for countries in ensuring availability of diagnostic tools. The EDL comprises 122 test categories including general and disease-specific tests. In the case of diabetes, tests included are those for glucose (urine dipsticks, glucometers and clinical chemistry and immunoassays), glycated haemoglogin (small analysers and clinical chemistry and immunoassays), and diabetic ketoacidosis (Electro-analytical method Handheld analyser).

Other guidance produced by the WHO, the Package of Essential Noncommunicable Disease Interventions (PEN), focuses on primary healthcare (PHC) [3] and includes in its “package” a set of diagnostic tools for the diagnosis and management of cardiovascular disease (CVD), diabetes and asthma, but not cancer. Within the PEN package, breast and cervical cancer are included, but with a view of “assessment and referral” of individuals [3]. In addition, it is important to note that WHO guidance in the area of diagnostic and monitoring focuses on facility-based tools and does not consider tools for individual’s self management of their condition.

However, both at facilities and for individuals, NCD diagnostic and monitoring tools have been found to be unavailable within health systems and/or unaffordable to individuals who have to pay for tests within the health system or purchase these tools for their self-monitoring. For example, for diabetes it was found that in Mali, Mozambique and Zambia urine test strips and blood glucose monitors (BGMs) were available in 54 % and 13 %, and 18 % and 21 %, and 61 % and 49 % of health facilities, respectively [4]. Costs of facility based testing, assuming one monthly blood glucose test, ranged from free in Nicaragua to US$ 27 per annum in Mali. For the use of personal meters, only wealthy individuals in low- and middle-income countries (LMIC) [4] and children benefitting from donation programs [5] had access to these devices. Similar cases of inequity may occur in other LMICs. To date, many studies have looked at the availability or affordability of these tools [4, 6, 7], but not necessarily if they are “technically” adapted to LMIC settings. This Commentary will present the current landscape and challenges with regards to diagnostic and monitoring tools for NCDs included in WHO’s guidance for PHC using the REASSURED criteria [8] in order to understand barriers to access to diagnostic tools, the complexities of these need to be assessed.

## The REASSURED Criteria

The REASSURED criteria (Table 1) are a set of characteristics developed for assessing diagnostic tools for communicable diseases (CD) and have been used for over a decade with the recent addition of three new attributes: real time connectivity, ease of specimen collection, and environmentally friendly [8] which allow for the key characteristics of diagnostic tools, specifically for LMICs, to be assessed. These include [8]:

**Table 1:**
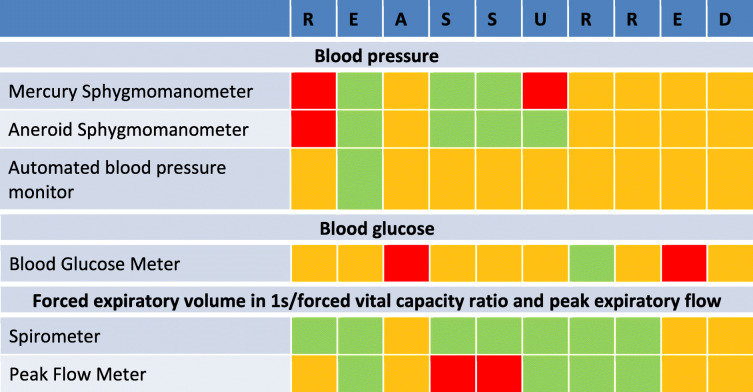
REASSURED criteria for diagnostic tests included in WHO PEN Package

This table presents each of the REASSURED criteria using a traffic light system with red: not at all; orange to a certain extent; and green yes

- Real time connectivity: Tests are connected and/or a reader or mobile phone is used to power the reaction and/or read test results to provide required data to clinicians and users.
- Ease of specimen collection: Tests should be designed for use with non-invasive specimens.
- Affordable: Tests are affordable to end-users and the health system.
- Sensitive: Avoid false negatives.
- Specific: Avoid false positives.
- User-friendly: Procedure of testing is simple — can be performed in a few steps, requiring minimum training.
- Rapid and Robust: Results are available to ensure treatment of patient at first visit (typically, this means results within 15 min to 2 h) and the tests can survive the supply chain without requiring additional transport and storage conditions such as refrigeration.
- Equipment free and environmentally friendly: Ideally the test does not require any special equipment or can be operated in very simple devices that use solar or battery power. Completed tests are easy to dispose and manufactured from recyclable materials.
- Deliverable to end-users: Accessible to those who need the tests the most.

## Applying the REASSURED Criteria to Noncommunicable disease diagnostic and monitoring tools

In order to present an overall view of the barriers to NCD diagnostics the REASSURED criteria were applied to tools used to diagnose and manage the NCDs included as part of the PEN package, namely CVD, diabetes and asthma based on a review of the literature.

For blood pressure different means of measurement exist, such as using a mercury or aneroid sphygmomanometer or an automated blood pressure monitor [9]. Mercury sphygmomanometers are seen as the gold standard technique for measuring blood pressure, with high sensitivity and specificity compared to aneroid and automatic monitors. However, this tool requires the most training of personnel. Overall, availability and affordability of blood pressure measuring devices are variable and all tools require additional equipment (i.e. either a stethoscope, batteries) and calibration [9–11]. All tools face similar issues with regards to robustness of the test.

BGMs are essential for the diagnosis and monitoring of diabetes. Compared to gold standard laboratory based tests, BGMs are not specific or sensitive [12]. Affordability and availability of BGM as a diagnostic and monitoring tool in health facilities as well as a monitoring tool for individuals are challenges [4]. Part of this cost is also due to the need for single-use strips that are only compatible with a given brand of meter as well as other consumables, such as lancets and batteries. Issues of robustness also exist as these tools can be affected by temperature and humidity [13]. It should also be noted that meters change frequently with an impact on which strips can be used.

Forced expiratory volume in 1s/forced vital capacity ratio (FEV_1_/FVC ratio: Tiffeneau-Pinelli index) is used in the diagnosis and management of asthma and chronic obstructive pulmonary disease (COPD) using a spirometer [14]. The peak flow rate can also be used for the diagnosis and follow-up of people with COPD and asthma. For the measurement of the peak expiratory flow (PEF), a peak flow meter can be used. Both spirometers and peak flow meters are used to determine lung capacity using different calculations. However, measurement error is higher for PEF [14]. Peak flow meters in general have poor sensitivity and specificity in diagnosing asthma and COPD and are mainly used for monitoring these conditions. Cost, availability and the need for consumables are the main barriers of access to spirometry [15]. In addition, health professional training can be seen as a secondary barrier as well as an absence of guidelines for both tests.

Details of the REASSURED criteria for all these tests are included in Table 1.

## Diagnostic tools for NCDs: more guidance is needed

Clearly more attention with regards to diagnostics as a key component of the health system is needed. Despite the existance of global guidance, the issue of access to diagnostics does not feature prominently on the global NCD agenda, in comparison for example to access to medicines. The EDL and PEN Package provide a list of tools that should be available, and the NCD GAP a target to be achieved. However, the target included in the NCD GAP focuses on availability and affordability and our review of the tools using the REASSURED criteria presents a wide range of challenges beyond price and availability, such as temperature stability, adaptability to various settings (e.g. at high altitude), need for training in order to perform and interpret the test, the need for maintenance and calibration, and for BGMs non-compatible meters and test strips. When used as self-monitoring tools by the individual at home they require training and skills to be delivered by capable health professionals. For NCDs, the EDL only includes tests where a sample needs to be taken and does not include blood pressure measuring devices or spirometers, which are also part of the equipment needed to diagnose and monitor patients.

## Conclusions

Access to diagnostic tests for NCDs does not necessarily result in their wide uptake and effective use and better outcomes for NCD management, and needs to be integrated in a health system wide response, including an understanding of key barriers that prevent sustainable access to health facilities, medicines, trained professionals and information, education and empowerment for people with the given conditions. Diagnostic tools have played an important role in addressing many CDs, such as HIV, TB and neglected tropical diseases. For NCDs a whole portfolio of diagnostic and monitoring tools are required throughout the continuum of care. These include tools for the initial diagnosis of the individual. For most NCDs the diagnostic test, blood pressure or blood glucose, needs to be assessed with a view of the overall risk of the individual, versus a “yes or no” diagnosis. For the ongoing follow-up and management of the individual this overall risk needs to be monitored as well as the need to ensure the availability of tests within facilities. In addition, for diabetes and hypertension part of the management for the individual may be done at home and includes the use of different self-monitoring tools. Then at regular intervals additional biological or physiological factors may need to be measured to assess disease progression. Finally, specialised tests carried out less frequently to detect complications also form part of the overall toolbox needed. The barriers identified need to be addressed to ensure better availability, affordability and uptake of adapted tools for LMIC contexts. Innovation in diagnostics is an opportunity to address barriers to access and adapt current tools to LMIC contexts. However, from the very outset, innovation should consider the REASSURED criteria to ensure that the tools developed meet the needs of LMICs and assist in addressing the global NCD challenge.

## Data Availability

The full report on which this article is based is available upon request to the authors.

## Abbreviations

BGM: Blood glucose monitors
CD: Communicable diseases
COPD: Chronic obstructive pulmonary disease
CVD: Cardiovascular disease
EDL: Model Essential Diagnostics List
EML: Model List of Essential Medicines (EML)
FEV_1_/FVC ratio: Forced expiratory volume in 1s/forced vital capacity ratio
LMIC: Low- and middle-income countries (LMIC)
NCD: Noncommunicable diseases
PEF: Peak expiratory flow
PEN: Package of Essential Noncommunicable Disease Interventions
PHC: Primary health care
UHC: Universal Health Coverage
WHO: World Health Organization
